# The impact of self-interviews on response patterns for sensitive topics: a randomized trial of electronic delivery methods for a sexual behaviour questionnaire in rural South Africa

**DOI:** 10.1186/s12874-017-0403-8

**Published:** 2017-08-17

**Authors:** Guy Harling, Dumile Gumede, Tinofa Mutevedzi, Nuala McGrath, Janet Seeley, Deenan Pillay, Till W. Bärnighausen, Abraham J. Herbst

**Affiliations:** 1 Academic Unit of Primary Care and Population Sciences and Department of Social Statistics and Demography, University of Southampton, Southampton, UK; 2 Africa Health Research Institute, School of Nursing & Public Health, University of KwaZulu-Natal, KwaZulu-Natal, South Africa; 3 Research Department of Epidemiology & Public Health, University College London, London, UK; 4 University of Southampton, Southampton, UK; 5 London School of Hygiene & Tropical Medicine, London, UK; 6 Division of Infection and Immunity, University College London, London, UK; 7 Institute of Public Health, University of Heidelberg, Heidelberg, Germany

**Keywords:** Randomized trial, Interview methods, Mixed-methods, Sexual behaviour, Single-paper meta-analysis

## Abstract

**Background:**

Self-interviews, where the respondent rather than the interviewer enters answers to questions, have been proposed as a way to reduce social desirability bias associated with interviewer-led interviews. Computer-assisted self-interviews (CASI) are commonly proposed since the computer programme can guide respondents; however they require both language and computer literacy. We evaluated the feasibility and acceptability of using electronic methods to administer quantitative sexual behaviour questionnaires in the Somkhele demographic surveillance area (DSA) in rural KwaZulu-Natal, South Africa.

**Methods:**

We conducted a four-arm randomized trial of paper-and-pen-interview, computer-assisted personal-interview (CAPI), CASI and audio-CASI with an age-sex-urbanicity stratified sample of 504 adults resident in the DSA in 2015. We compared respondents’ answers to their responses to the same questions in previous surveillance rounds. We also conducted 48 cognitive interviews, dual-coding responses using the Framework approach.

**Results:**

Three hundred forty (67%) individuals were interviewed and covariates and participation rates were balanced across arms. CASI and audio-CASI were significantly slower than interviewer-led interviews. Item non-response rates were higher in self-interview arms. In single-paper meta-analysis, self-interviewed individuals reported more socially undesirable sexual behaviours. Cognitive interviews found high acceptance of both self-interviews and the use of electronic methods, with some concerns that self-interview methods required more participant effort and literacy.

**Conclusions:**

Electronic data collection methods, including self-interview methods, proved feasible and acceptable for completing quantitative sexual behaviour questionnaires in a poor, rural South African setting. However, each method had both benefits and costs, and the choice of method should be based on context-specific criteria.

**Electronic supplementary material:**

The online version of this article (doi:10.1186/s12874-017-0403-8) contains supplementary material, which is available to authorized users.

## Background

There has long been concern that the measurement of sexual behaviour is fraught with potential biases [1, 2]. In cross-section, there is a high likelihood that individuals will be affected by a desire to provide socially desirable responses. This social desirability bias may lead to over-reporting (e.g. men reporting numbers of sexual partners) or under-reporting (e.g. women reporting numbers of sexual partners) [3]. Additionally, recall of behaviour in the past is likely to suffer from unintentional errors which are at best random and at worst also affected by social desirability.

Longitudinally, there are additional concerns, all of which apply to both research on sexual behaviour and other outcomes. First, individuals may learn how to respond in order to minimize response burden, e.g. reporting fewer partners when each partner triggers a follow-up set of questions [4, 5]. Second, socially desirable responses may change over calendar time (e.g. after a publicity campaign promoting condom use, reported condom use levels may rise) or based on lifecourse stage (e.g. increasing self-reported age at first sex by the same individuals over time [6]). Third, the composition of open cohorts may change of over time, including in ways associated with behaviour (e.g. loss to HIV-related mortality). Such changes may mean that apparent trends reflect a combination of intra-and inter-respondent behaviour change [7]. These longitudinal effects may obscure actual changes in sexual behaviour over time, limiting the power of cohort data in inferring programme impact on actual behaviour.

Some of these potential biases may be tempered by using self-interview techniques. In self-interviews, instead of the interviewer asking questions and writing down responses, the respondent completes the form. A common format for self-interviews is the computer-assisted self-interview (CASI) where a computer programme leads the respondent through the questionnaire. This can be coupled with a headphone set to allow for audio-computer-assisted self-interviews (ACASI), which is particularly helpful in lower-literacy populations [8]. Any form of CASI, however, requires *form literacy*, i.e. the ability to navigate the questionnaire [9]. When computer-based this includes computer literacy; when paper-based respondents need to be able to interpret and follow skip patterns and other instructions.

A number of past studies have compared self-interview to face-to-face techniques. These include comprehensive reviews of sexual behaviour in low and middle-income countries [10, 11] and worldwide [12]. A further worldwide meta-analysis compared paper- and computer-based self-completed interviews [13]. No one method appears to be universally best, although on average sensitive behaviours appear to be reported more often during self-interviews, at least when first introduced [14]. Self-interview methods sometimes improve the rate of reporting of socially undesirable behaviours (e.g. number of sexual partners, forced sex [11, 15]) and decrease item non-response rates [16]. However, they also increase the level of internally inconsistent responses [17].

Interviewer-led interviews can be affected by interviewer-related variability in response [18]. However the required interaction with the interviewer sometimes leads to increased willingness to reveal highly sensitive answers [19], and may be particularly useful for complex or ambiguous questions (e.g. concurrency). Qualitative evidence suggests that respondents are more willing to accurately report sensitive topics in self-interviews [10], and respondents report that self-interview methods are preferable for sexual matters [20, 21]. Nevertheless, recent experiments using biomarkers found little difference in validity between face-to-face and self-interview arms [22, 23].

In the context of a long-running, paper-based longitudinal surveillance programme in rural South Africa, consideration has been given to how to improve questionnaire delivery. We therefore conducted a randomized trial with mixed methods evaluation of the feasibility and acceptability of using electronic methods to administer sexual behaviour questionnaires. We measured overall and item non-response rates, time taken to conduct the interviews and how the new methods were viewed by respondents and field staff.

## Methods

This electronic delivery methods study (“EDM”) compared four methods for delivering a questionnaire on sexual behaviour to participants. Research interviews can be considered to be any interaction between an interviewer and a respondent, in which questions are asked with the aim of eliciting information. Such interviews may use close-ended questions in a questionnaire format to capture structured information. Often such questions require responses that fit into one of a number of pre-determined categories (e.g. “have you ever had sex”) or are numeric (e.g. “how many sexual partners have you had in your lifetime”). Alternatively, they may require short responses (e.g. “which town did you grow up in”). Interviews can also use open-ended questions intended to elicit less structured responses (e.g. “how does going to church make you feel”). Such open-ended questions can be pre-scripted, or allowed to arise spontaneously as follow-up questions during the interview process. The EDM interview consisted of a structured, largely quantitative questionnaire with open-ended “cognitive interview” questions embedded between sections. The cognitive interview questions were intended to help us better understand responses to the close-ended questions. This interview was conducted on a single occasion at the home of the respondent.

The four methods we used to conduct our structured, quantitative questionnaire were: (1) Paper and pen interview (PAPI): the interviewer asks the questions and writes responses onto a paper form. (2) Computer-assisted personal interview (CAPI): the interviewer asks the questions and enters the responses into a portable tablet computer. (3) Computer-assisted self-interview (CASI): the respondent reads questions on the tablet and enters the responses themselves. (4) Audio Computer-assisted self-interview (ACASI): the respondent reads or listens using headphones to the questions on the tablet and enters the responses themselves. These were grouped into interviewer-led arms (PAPI and CAPI) and respondent-led arms (CASI and ACASI).

The study was conducted in August and September 2015 in the Somkhele demographic surveillance area (DSA) of the Africa Health Research Institute (AHRI). The DSA is a ~ 435 km^2^ area in the uMkhanyakude district of KwaZulu-Natal province. The DSA has been under semi- or tri-annual household demographic surveillance since 2000, including annual individual health questionnaires since 2003 [24]. This health surveillance questionnaire consists of closed-ended or very short text quantitative questions, and contains sections on general health (chronic conditions, healthcare utilization), sexual health (marital status, contraception, paternity/maternity, circumcision) and sexual behaviour, including partner-specific behaviour covering up to three partners from the past 12 months [25]. The DSA contains one urban area (KwaMsane) but is otherwise rural. There are ~11,000 households in the DSA, and any resident household member aged 15 and over who can consent is eligible for the health questionnaire. All surveillance questionnaires are conducted as PAPI.

At the beginning of 2015, 36,336 individuals were listed as potentially eligible for health surveillance in that year. Of these, 10.9% had died, migrated or their household was dissolved prior to interview and were considered no longer eligible by the time they were approached between February and April 2015. Of those still eligible, a further 7.2% were not contactable. Of those contacted, 5.4% were unable to provide informed consent, and a further 1.2% were too sick to participate. Of those contacted and capable of consent, 54.8% consented to be interviewed. Of those who consented, 49.6% (27.2% of all eligible individuals) answered any of the sexual behaviour questions. Literacy rates in this area are high; in 2014 77.9% of residents aged 18–49 had attended secondary school and 45% had reached the final year of secondary school.

### Study design

For the quantitative questionnaire, we drew a random stratified sample of 504 individuals aged 18 and over who were eligible for health surveillance questionnaires in the first 14 weeks of surveillance in 2015, i.e. were resident members of a DSA household at the previous household surveillance visit (conducted between August and December 2014). We expected to interview 75% of sampled individuals (allowing for migration and non-consent). We therefore expected this sample size to provide 80% power to see a rise in the proportion of individuals reporting more than one partner in the past 12 months from 3% in 2013 health surveillance to the national level of 12.5% [26], when comparing respondent- and interviewer-led techniques.

The sample consisted of equal numbers from four of the 23 *izigodi* (traditional Zulu community areas, singular *isigodi*) within the DSA: one urban; one peri-urban; and two rural locations. Within each *isigodi* we further stratified the sample into six equal sets of 21 by gender and three age categories: 18–29, 30–49 and over 50. We made two attempts to contact each selected individual at their place of residence. In line with existing DSA procedures, reasons for no longer being eligible were: (i) death; (ii) dissolution of the household; (iii) out-migration from the household. All those contacted were interviewed unless they were incapable of providing informed consent or declined to interview.

The EDM questionnaire contained seven sections. Many of the questions we used were the same as those asked in annual surveillance questionnaires, but we also included new questions that we expected to be particularly sensitive to answer in this setting. We endeavoured to keep our question wording as close as possible to that used in annual surveillance questionnaires, although we did retranslate the text for this study. The first section, on marital status, was asked by the interviewer in all trial arms. Three sections were gender-specific: pregnancy and contraception (women only); paternity (men only); circumcision (men only). These first four sections contained exactly the same questions as the surveillance questionnaire.

Section five covered general sexual history, including numbers of partners and use of condoms. This section contained all surveillance questionnaire questions, with additional new questions on numbers of sexual acts and regularity of condom use in the past 4 weeks. Section six asked about partner-specific sexual history on up to three most-recent partners within the past 12 months. The final section asked about lifetime involvement in high-risk sexual behaviours, i.e. exchange sex, anal sex, same-sex involvement and forced sex. All of these questions were new. In this analysis we focus on the last three sections of the questionnaire covering sexual behaviour (general and partner-specific sexual history), since these are the sections most likely to be affected by social desirability bias and non-response [27].

After the interviewer-led marital status section, individuals allocated to self-interview arms (CASI or ACASI) were provided with an additional brief section introducing them to the tablet software. This training section included examples of different question types (e.g. numeric, multiple-choice, text entry) using non-sensitive, non-health questions. Respondents were informed that this was a training section and that their responses in the section would not be analysed. All arms required all questions to be answered before progressing, and all questions included a “Prefer not to answer” option, although this was not explicitly presented to respondents in the interviewer-led arms. The questionnaire was programmed in OpenDataKit [28], a free open-source software, and all commands and questions were translated into isiZulu and the translations piloted within the study team. While every respondent was allocated to a specific study arm, those in self-interview arms were offered the opportunity to conduct the questionnaire as a CAPI if they preferred, and were also told they could ask for assistance from the interviewer at any time; the level of assistance provided was recorded at the end of the interview.

Within each study arm we randomly selected 12 individuals to be invited to participate in a cognitive interview [29, 30]. Cognitive interviewing is a qualitative method for helping to identify potential sources of error in questionnaire responses. The method focuses explicitly on understanding the cognitive processes used by respondents in answering research questions in four stages. First, *question comprehension*: what does the respondent believe the question to be asking. Second, *retrieval of relevant information*: what types of information does the respondent need to recall and what strategies do they use to answer the question. Third, *decision process*: does the respondent want to tell the truth and how much mental effort is dedicated to answering the question accurately. Fourth, *response process*: can the respondent match their internally generated answer to the question categories. Questions were open-ended and we used the verbal probing approach based on initial scripted probes followed by spontaneous follow-up probes to unpack responses. The approach has been used previously in sexual behaviour questionnaire development [31, 32].

After each of the seven sections of the questionnaire, we used both broad and question-specific cognitive interview probes. We additionally asked a set of overarching questions about the interview process after all quantitative data collection was complete in order to understand the overall acceptability of using electronic data collection methods, both in-and-of-themselves and relative to past paper-based approaches. These cognitive interviews were transcribed and translated into English. We continued to invite allocated individuals to participate in cognitive interviews until the qualitative interviewers in discussion with the qualitative coordinator agreed that saturation had been reached.

After completing all data collection for the trial, we conducted a group discussion with all six interviewers to gather information on the lessons they had learned from the study. Specifically, we asked about interactions with the local community, which questions respondents found problematic and about the experiences of fieldworkers and respondents in using electronic tablets for data collection.

### Analytic design

We first describe rates of contact and consent by arm, as well as interview duration. Our primary quantitative outcomes of interest are rates of: (i) overall response; (ii) item response for sexual behaviour questions; (iii) affirmative responses to sexual behaviour questions. Our primary comparison was an intention-to-treat (ITT) analysis between interviewer- and respondent-led arms (to protect against non-random switching from self-interview to CAPI arms); as a secondary analysis we conducted an As Treated (AT) analysis. Differences were examined using *χ* ^2^ tests for binary outcomes and Kruskal-Wallis tests for continuous and ordinal outcomes. We present effect size estimates using $\phi =Z/\sqrt{N}$ for *χ* ^2^ tests and $r=\sqrt{\chi^2/N}$ for Wilcoxon rank-sum tests. Both measures provide an estimate of the proportion of variance seen that is due to correlation between study arms and the response variable of interest.

To summarize our findings we also conducted a single-paper meta-analysis (SPM) of non-response by arm for the 24 sexual behaviour questions, and affirmative proportions for all 15 binary outcome questions. We used a restricted maximum-likelihood estimator in a random-effects model to estimate the mean difference in proportions of either item non-response or affirmative response, comparing interviewer- and respondent-led arms. We further estimated between-question heterogeneity responses across study arms using *I* ^2^, the percentage of observed variance due to variance in true effect sizes rather than chance [33]. Our working hypothesis was that respondent-led arms would have the greatest increase in response rates for the most sensitive questions. A priori, we expected these to include questions about partner numbers, concurrent relationships, explicitly exchanging sex for goods or money, having anal sex (highly stigmatized in South Africa [34]), same-sex attraction and forced sex. We therefore ran a third SPM for just the seven binary outcomes for highly sensitive questions.

In addition to conducting cross-sectional analysis, we also compared individuals’ responses in this trial to their most-recent responses in a surveillance questionnaire. This supplementary analysis aimed to evaluate to what extent results seen in the EDM trial reflected the trial environment itself: i.e. if those in the interviewer-led arms responded differently in the EDM versus in surveillance.

Finally, we assessed the acceptability and feasibility of answering questions relating to sexual health, and the benefits or drawbacks of using electronic delivery methods, using data from the cognitive interviews. We used the Framework approach to derive a case-and-theme structure from the cognitive interview data [35], and focused on key prompts relating to each sexual behaviour section and to the overall questionnaire – including a comparison of their experiences of the EDM study compared to past annual surveillance (prompts listed in Additional file 1: Content S1). Initial coding was conducted by GH and DM who compared selected scripts which they had coded separately to ensure consistent codes were used.

## Results

**Fig. 1:**
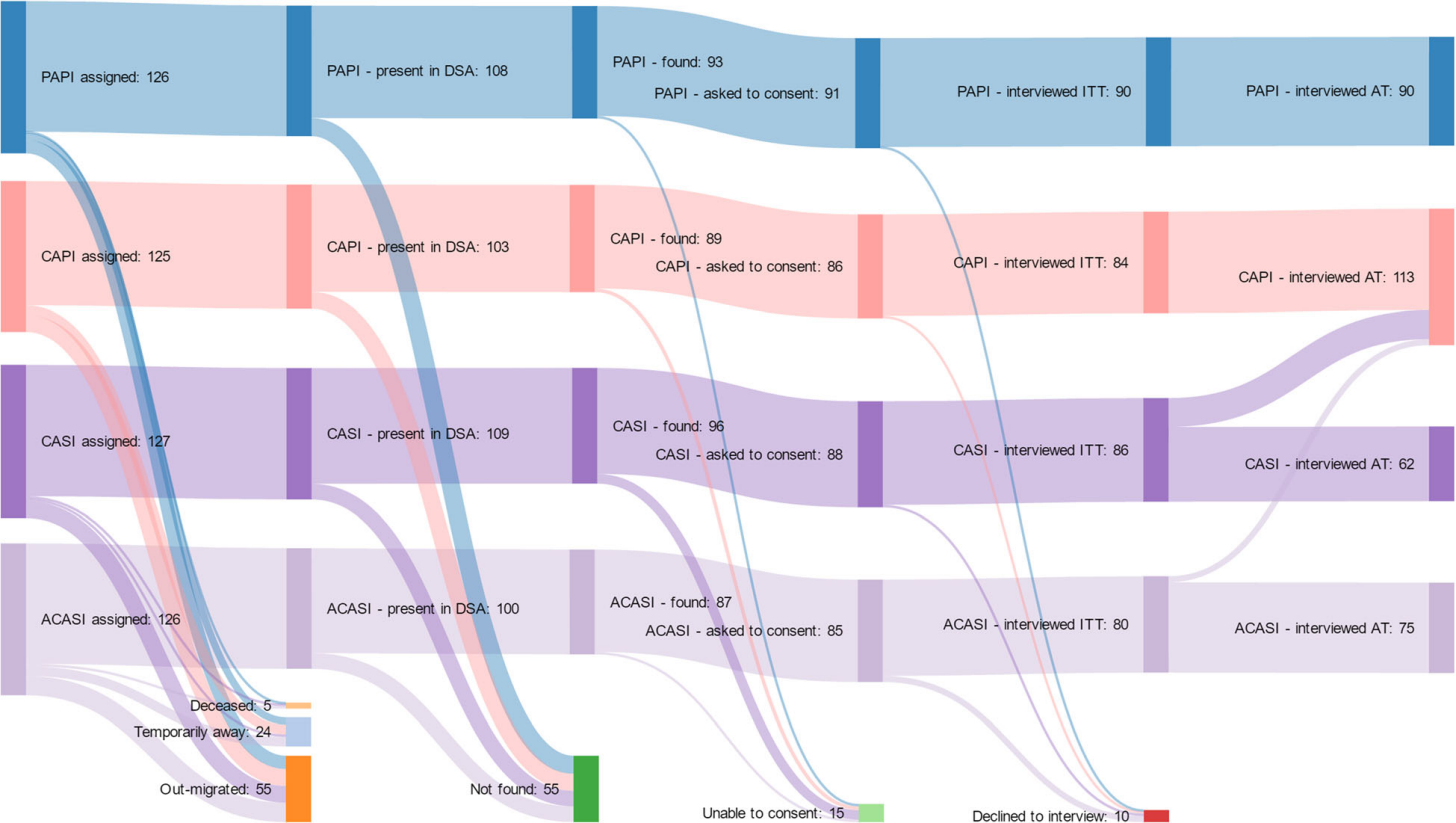
Sankey diagram of study outcomes for sampled individuals. Data underlying this figure are shown in Additional file 1: Table S1

The flow of the 504 potential respondents sampled through the trial is shown in Fig. 1. 84 (16.7%) of sampled individuals were not in the DSA, and thus no longer eligible, and further 55 (10.9%) could not be contacted within the study period. Amongst the 365 individuals contacted, 15 (3.0%) of individuals were unable to provide informed consent and 10 more (2.0%) declined to participate. Each arm was balanced by design on gender, age and location, and there were no statistical differences in the number of individuals being contacted or consenting to participate by arm (Table 1). Older and non-urban individuals were significantly more likely to be contacted, but there were no differences in willingness to participate once contacted.

**Table 1:** Respondent characteristics by response status and intention-to-treat arm

	PAPI	CAPI	CASI	ACASI	Z	*p* ^†^	Total	%	Z	*p* ^‡^
A. Full sample (*n* = 504)										
Gender										
Male	61	62	65	64			252			
Female	65	63	62	62	0.24	0.97	252			
Age										
18–29	42	42	39	39			162			
30–49	41	41	42	44			168			
≥ 50	43	42	46	43	0.56	1.00	174			
Location										
Urban	33	31	30	32			126			
Peri-urban	31	30	34	31			126			
Rural	62	64	63	63	0.46	1.00	252			
B. Contacted sample (*n* = 355)										
Gender										
Male	49	46	50	45			190	0.75		
Female	44	43	46	42	0.02	1.00	175	0.69	2.24	0.14
Age										
18–29	24	25	29	25			103	0.64		
30–49	33	29	30	29			121	0.72		
≥ 50	36	35	37	33	0.62	1.00	141	0.81	12.8	0.002
Location										
Urban	22	20	14	18			74	0.59		
Peri-urban	21	26	28	23			98	0.78		
Rural	50	43	54	46	3.86	0.70	193	0.77	15.8	<0.001
C. Interviewed sample (*n* = 340)										
Gender										
Male	48	44	45	44			181	0.95		
Female	42	40	41	36	0.15	0.99	159	0.91	2.77	0.10
Age										
18–29	24	24	25	23			96	0.93		
30–49	31	28	26	26			111	0.92		
≥ 50	35	32	35	31	0.45	1.00	133	0.94	0.69	0.71
Location										
Urban	21	19	13	16			69	0.93		
Peri-urban	20	24	24	22			90	0.92		
Rural	49	41	49	42	3.20	0.78	181	0.94	0.39	0.82

*PAPI* Paper and pen interview, *CAPI* computer-assisted self-interview, *CASI* computer-assisted personal interview, *ACASI* audio computer-assisted personal interview. All non-test values are counts

^†^ ${\chi}_3^2$ tests for gender, ${\chi}_6^2$ tests otherwise, for difference across arms and stratification cells by each stratifying variable

^‡^ ${\chi}_1^2$ tests for gender, ${\chi}_2^2$ tests otherwise, for difference in proportion of allocated individuals being contacted (panel B) and the proportion of contacted individuals interviewing (panel C) across levels of each stratifying variable

Of the 166 consenting respondents who were assigned to the CASI or ACASI arms, 29 (17%) did not complete the questionnaire as a self-interview (24/86 CASI [6.3%] vs 5/80 ACASI [27.9%]; ${\chi}_1^2=13.5$, *p* < 0.001). The most common reasons given for requesting CAPI rather than a self-interview were: inability to read or write (*n* = 15); eyesight problems (*n* = 9); and dislike of computers (*n* = 2). The proportion of individuals who declined self-interviews rose with age, from 2.1% amongst 18–29 year olds to 19.2% amongst 30–49 year olds to 27.3% amongst over 50 year-olds (Cuzick non-parametric trend test: *Z* =  − 3.4, *p* = 0.001), but was not significantly different by gender (female: 19.1%; male: 15.6%, ${\chi}_1^2=0.4$, *p* = 0.55).

**Fig. 2:**
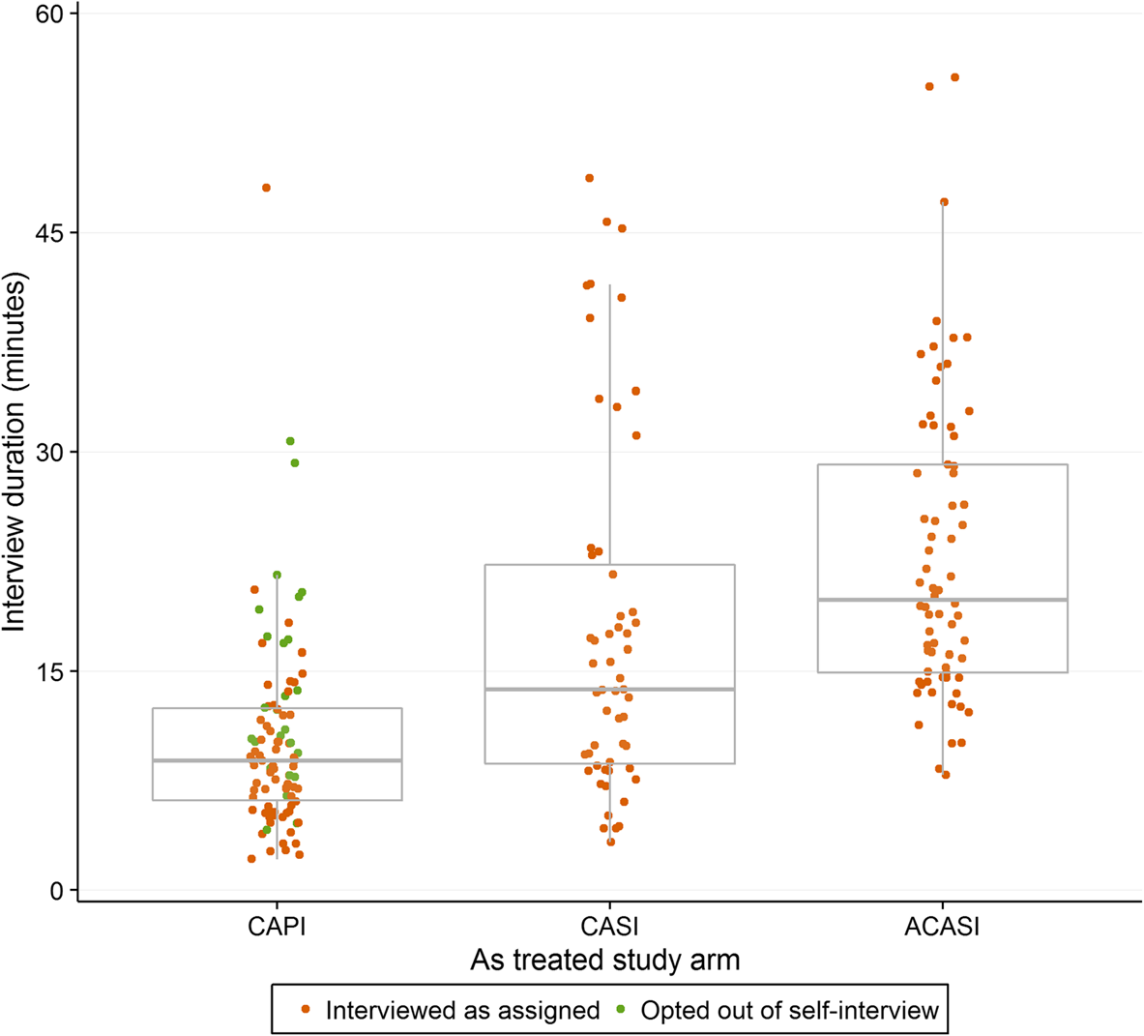
Interview duration for tablet computer study arms. *N* = 219. All durations measured as end of interview time minus start of interview time, so no data is presented for the Paper and Pen Interview (PAPI) arm. Five individuals with a reported interview length of greater than 60 min (CAPI: 271 min; CASI: 157 min; ACASI: 63, 94 and 357 min; the 357 min interview was opted-out to CAPI), and all 20 individuals completing cognitive interviews, on the understanding that these interviews had been interrupted, are not shown

Across the three computer-based arms (CAPI, CASI, ACASI), interview duration varied systematically by arm among the 224 non-cognitive interview respondents (Fig 2). Under ITT, median duration was 8.3 min (interquartile range (IQR) 5.4–11.70) in the CAPI arm, 13.7 (IQR 13.7–20.1) in the CASI arm and 19.9 (IQR 14.6–30.9) in the ACASI arm; all distributions were right-skewed (skewness: 6.9; 5.0; 7.5). These differences were significant and moderately sized using a Wilcoxon rank-sum test: CAPI vs. CASI, Z = 4.9, *p* < 0.001, *r* = 0.40; CASI vs. ACASI, Z = 4.1, *p* < 0.001, *r* = 0.33. AT analysis results were qualitatively similar, although the 26 individuals opting-out of self-interview arms and into CAPI took a median of 12.4 min (IQR 12.4–19.2), significantly longer than those who did not opt-out (Wilcoxon Z = 2.6, *p* = 0.009, *r* = 0.21). There were no significant differences, either overall or within study arms, by age group or respondent gender.

Item non-response rates were generally higher in self-interview arms (Tables 2, 3, 4). In meta-analysis, self-interview respondents were significantly more likely to avoid responding to questions (Additional file 1: Figure S2). The mean percentage of respondents declining to answer was 4.4% in the interviewer-led arms versus 6.5% in the self-interview arms (mean difference: 2.1%, 95% confidence interval: 0.1–3.3%). However, this difference should be treated with caution given the high level of heterogeneity across questions: non-response was significantly (up to 10 percentage points) higher in self-interviews for several questions relating to respondents’ most-recent partner, but (non-significantly) lower for a range of other questions. Quantitatively, heterogeneity of effects for non-response was estimated to be very high (*I* ^2^=88.4, 95%CI: 85.4–90.7%).

**Table 2:** Item response rates for general sexual behaviours

	ITT analysis							AT analysis						
	PI arms		SI arms		*Z*	*p*	*φ*	PI arms		SI arms		*Z*	*p*	*φ*
*Total number of respondents*	*174*		*166*					*203*		*137*				
Ever had sexual intercourse														
Yes	165	95%	159	94%	0.47	0.49	0.04	194	97%	130	95%	0.00	0.99	0.00
Declined to answer	0	0%	1	<1%	1.05	0.31	0.06	0	<1%	1	<1%	1.49	0.22	0.07
Age at first sex														
Median (IQR)	18	[17–20]	18	[16–20]	1.87	0.17	0.07	18	[17–20]	18	[16–20]	2.26	0.13	0.08
Declined to answer	30	17%	25	15%	0.35	0.56	0.03	35	18%	20	15%	0.39	0.53	0.03
Lifetime number of sexual partners														
Median (IQR)	2	[1–5]	2	[1–4]	0.07	0.79	0.01	2	[1–4]	3	[1–5]	0.44	0.51	0.04
Declined to answer	31	18%	28	17%	0.08	0.78	0.01	33	16%	26	19%	0.47	0.49	0.04
Number of partners in past 12 months														
Median (IQR)	1	[0–1]	1	[1–1]	4.13	**0.04**	0.11	1	[0–1]	1	[1]	9.10	**0.003**	0.16
Declined to answer	16	9%	25	15%	3.40	0.07	0.10	18	9%	23	17%	6.17	**0.01**	0.13
Number of sex acts with MRP in past 4 weeks ^a^														
Median (IQR)	2	[1–3]	2	[1–4]	0.11	0.74	0.02	2	[1–4]	1.5	[1–4]	1.41	0.23	0.06
Declined to answer	18	10%	31	19%	9.88	**<0.001**	0.17	21	10%	28	20%	13.8	**<0.001**	0.20
Frequency of condom use in past 4 weeks ^a^														
Never	14	17%	20	30%				16	55%	18	69%			
Sometimes	8	10%	5	7%				9	29%	4	20%			
Most of the time	14	17%	8	12%				14	16%	8	16%			
Always	43	52%	31	46%	3.80	0.28		53	50%	21	28%	6.28	**0.10**	
Declined to answer	3	2%	3	2%	0.06	0.80	0.01	3	1%	3	2%	0.51	0.47	0.04
Any concurrent relationships today														
Yes	5	3%	12	6%	3.39	0.07	0.10	8	5%	9	6%	1.19	0.28	0.06
Declined to answer	11	6%	11	7%	0.01	0.92	0.01	13	6%	9	7%	0.01	0.92	0.01
Any concurrent relationships in past 12 months														
Yes	8	8%	12	11%	1.35	0.25	0.06	11	13%	9	11%	0.22	0.64	0.03
Declined to answer	1	<1%	2	1%	0.43	0.51	0.04	1	<1%	2	1%	0.87	0.35	0.05

*ITT* Intention-to-treat, *AT* As-treated, *PI* personal interview arms (PAPI, CAPI), *SI* self-interview arms (CASI, ACASI), *IQR* inter-quartile range, *MRP* most recent partner. Z test statistics compare PI to SI arms. In each pair the upper value is a ${\chi}_1^2$ comparison of the proportion of affirmative responses amongst valid responses for binary outcomes and a non-parametric Kruskall-Wallis test with *k* − 1 degrees of freedom for continuous and ordinal variables. The lower value is a comparison of item non-response rates using a ${\chi}_1^2$ test. *φ is the effect size associated with the relationship between interview arm and the outcome of interest.* ^a^ These questions had not been asked in recent annual surveillance questionnaires

**Table 3:** Item response rates for sexual behaviour questions not previously used in the surveillance

	ITT analysis							AT analysis						
	PI arms		SI arms		*Z*	*p*	*φ*	PI arms		SI arms		*Z*	*p*	*φ*
*Total number of respondents*	*174*		*166*					*203*		*137*				
Given gifts in past 12 months														
Yes	79	45%	67	40%	0.46	0.50	0.04	95	47%	51	37%	2.04	0.15	0.08
Declined to answer	8	5%	13	8%	1.53	0.22	0.07	9	4%	12	9%	2.64	0.10	0.09
Given gifts to get sex in past 12 months ^a^														
Yes	10	13%	12	15%	0.85	0.36	0.05	12	13%	10	20%	1.20	0.27	0.06
Declined to answer	0	0%	1	1%	1.19	0.28	0.06	1	0%	0	0%	0.54	0.46	0.04
Received support in past 12 months														
Yes	60	34%	63	34%	1.11	0.29	0.06	69	34%	54	39%	2.23	0.13	0.08
Declined to answer	6	3%	14	8%	3.81	**0.05**	0.11	7	3%	13	9%	5.39	**0.02**	0.13
Had sex to get support in past 12 months ^a^														
Yes	1	2%	3	5%	0.97	0.33	0.05	1	1%	3	6%	1.68	0.20	0.07
Declined to answer	0	0%	1	1%	0.96	0.33	0.05	0	0%	1	1%	1.29	0.26	0.06
Ever had anal sex														
Yes	5	3%	6	3%	0.18	0.67	0.02	5	2%	6	4%	0.95	0.33	0.05
Declined to answer	10	6%	13	8%	0.59	0.44	0.04	14	7%	9	7%	0.01	0.91	0.01
Ever had sexual experience with same gender														
Yes	2	1%	5	3%	1.55	0.21	0.07	4	2%	3	2%	0.03	0.86	0.01
Declined to answer	3	2%	7	4%	1.85	0.17	0.07	4	2%	6	4%	1.66	0.20	0.07
Ever had someone try to make you have sex against your will														
Yes	7	4%	10	5%	0.88	0.35	0.05	10	5%	7	5%	0.03	0.87	0.01
Declined to answer	0	0%	7	4%	7.49	**0.01**	0.15	1	0%	6	4%	6.13	**0.01**	0.13

*ITT* Intention-to-treat, *AT* As-treated, *PI* personal interview arms (PAPI, CAPI), *SI* self-interview arms (CASI, ACASI), *IQR* inter-quartile range. Z test statistics compare PI to SI arms. In each pair the upper value is a comparison of the proportion of affirmative responses amongst valid responses and the lower value is a comparison of item non-response rates using a ${\chi}_1^2$ test. *φ is the effect size associated with the relationship between interview arm and the outcome of interest.* ^a^ These questions were only asked of those responding “Yes” to the preceding question. None of the questions in this table had previously been asked in annual surveillance questionnaires

**Table 4:** Item response rates for partner-specific sexual behaviours with most-recent sexual partner

	ITT analysis							AT analysis						
	PI arms		SI arms		*Z*	*p*	*φ*	PI arms		SI arms		*Z*	*p*	*φ*
*Total number of respondents*	*154*		*131*					*181*		*104*				
How first met ^a^														
Known since childhood	28	18%	20	15%				32	17%	16	15%			
Through a mutual friend	4	3%	7	5%				5	2%	6	6%			
At work, school, university	47	31%	35	27%				53	29%	29	28%			
Online	1	1%	2	2%				1	1%	2	2%			
At a sporting event	0	0%	2	2%				1	1%	1	1%			
At a religious event	10	6%	13	10%				14	8%	9	9%			
At a friend/relatives’	4	3%	4	3%				5	3%	3	3%			
At a shebeen or club	0	0%	1	1%				1	1%	0	0%			
At the river	9	6%	6	5%				12	6%	3	3%			
On the street	17	11%	4	3%				17	9%	4	4%			
In town	12	8%	3	2%				13	7%	2	2%			
Other	16	10%	19	15%				20	11%	15	14%			
Declined to answer	6	4%	15	11%	5.92	**0.01**	0.14	7	4%	14	13%	8.91	**0.003**	0.18
Relationship at last sex ^b^														
Conjugal relationship	34	22%	20	15%				44	18%	10	6%			
Steady relationship	64	42%	34	26%				72	32%	26	17%			
Ex-steady relationship	47	31%	48	37%				54	30%	41	39%			
Known to one-another	2	1%	9	7%				4	2%	7	7%			
Not known to one-another	1	1%	2	2%				1	1%	2	2%			
Declined to answer	5	3%	18	14%	10.5	**0.001**	0.19	5	3%	18	17%	18.8	**<0.001**	0.26
Still in a sexual relationship														
Yes	102	66%	83	63%	0.07	0.79	0.02	117	35%	68	65%	1.37	0.24	0.07
Declined to answer	2	1%	10	8%	7.04	**0.01**	0.16	2	1%	10	10%	11.9	**0.001**	0.20
Age difference of partner														
Median (IQR), women	4	[0–7]	4	[0–8]	0.03	0.85	0.01	4	[0–7]	3	[0–8]	0.09	0.76	0.02
Median (IQR), men	−3	[−5.5–0]	−3	[−6–0]	0.29	0.59	0.03	−3	[−6–0]	-3	[−5.5–0]	0.03	0.87	0.01
Declined to answer	13	8%	4	3%	3.25	0.07	0.11	14	8%	3	3%	2.45	0.12	0.09
Partner a household member														
Yes	66	43%	57	44%	0.37	0.54	0.04	82	25%	41	39%	0.12	0.73	0.02
Declined to answer	2	1%	10	8%	7.04	**0.01**	0.16	2	1%	10	10%	11.9	**0.001**	0.20
Ever used a condom														
Yes	72	47%	58	44%	0.00	1.00	0.00	78	23%	52	50%	2.8	0.09	0.10
Declined to answer	0	0%	7	5%	8.44	**0.004**	0.17	0	0%	7	7%	12.5	**<0.001**	0.21
Frequency of condom use														
Never	21	14%	25	19%				24	12%	22	17%			
Sometimes	25	16%	16	12%				28	14%	13	10%			
Most of the time	26	17%	15	11%				26	10%	15	9%			
Always	72	47%	56	43%	3.33	0.34	0.11	78	43%	50	48%	2.52	0.47	0.09
Declined to answer	0	0%	2	2%	2.52	0.11	0.09	0	0%	2	2%	3.05	0.08	0.10
Condom use at first sex														
Yes	44	31%	44	34%	0.05	0.82	0.01	47	15%	41	39%	3.99	0.05	0.12
Declined to answer	15	10%	8	6%	1.71	0.19	0.08	15	8%	8	8%	0.11	0.74	0.02
Condom use at last sex														
Yes	42	29%	43	33%	0.44	0.51	0.04	48	15%	37	36%	2.25	0.13	0.09
Declined to answer	13	8%	9	7%	0.37	0.54	0.04	13	7%	9	9%	0.12	0.72	0.02

*ITT* Intention-to-treat, *AT* As-treated, *PI* personal interview arms (PAPI, CAPI), *SI* self-interview arms (CASI, ACASI), *IQR* inter-quartile range. Z test statistics compare PI to SI arms. In each pair the upper value is a ${\chi}_1^2$ comparison of the proportion of affirmative responses amongst valid responses for binary outcomes and a non-parametric Kruskall-Wallis test with *k* − 1 degrees of freedom for continuous and ordinal variables. The lower value is a comparison of item non-response rates using a ${\chi}_1^2$ test. *φ is the effect size associated with the relationship between interview arm and the outcome of interest.* ^a^ This question had not been asked in recent annual surveillance questionnaires. ^b^ This question had been asked in recent annual surveillance questionnaires, but the categories of responses were more precise in this trial

**Fig. 3:**
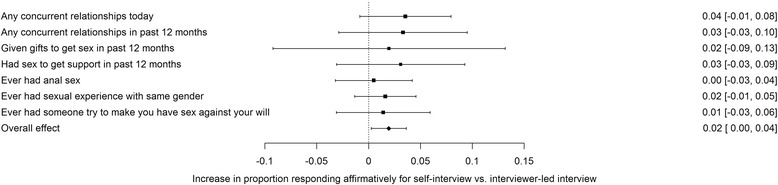
Single-paper meta-analysis of most sensitive binary response questions. Size of point estimates is in proportion to the log of the number of respondents for each question. Values at right are means and 95% confidence intervals

Amongst those who answered questions, in only a few cases were there significant differences between interviewer-led and self-interview arms (Tables 2, 3, 4). However, meta-analysis highlighted that self-interview respondents were more likely answer affirmatively to seven binary highly sensitive questions: mean percentage answering yes: 6.1% vs 4.2% for interviewer-led arms (Fig. 3). This difference was relatively small in absolute terms, but statistically significant (mean: 1.9%, 95% confidence interval [CI]: 0.3–3.6%). Heterogeneity of effects was estimated to be moderate (***I*** ^2^=65%, 95% CI: 36–81%), although all effects were in the same direction. When we considered all 15 binary questions, the results were highly heterogeneous and no significant association was seen (Additional file 1: Figure S3). Effect sizes for both item non-response and affirmative responses were small to moderate, with a highest value of *ϕ* = 0.21 and mostly with values <0.10.

Our supplementary analysis comparing respondents’ EDM questionnaire responses to their prior surveillance questionnaire is presented in Additional file 1: Content S2. We did not find any significant differences either in surveillance responses or changes between last surveillance response and EDM response across EDM arms. Questions that should have time-invariant responses (e.g. age of sexual debut) did not significantly change between surveillance to EDM questionnaires.

### Cognitive interviews

#### Acceptability and feasibility of sexual behaviour questions

In this area where sexual health surveillance has been conducted for over 10 years, few respondents found the topics covered unacceptable or difficult. Almost all respondents reported positive feelings towards answering sexual history questions that they had seen before, using terms such as ‘happy’, ‘no problem’, ‘comfortable’, ‘alright’ and ‘okay’. Difficulties in responding to sexual health questions revolved around question complexity – either due to long periods of recall or unclear question phrasing – or the inclusion of new topics that some respondents were not expecting:

“*I don’t know how many different people I have had sex with in my lifetime. I am unable to count. When one grows up, you have sexual partners here and there. I did not save them in my memory because I didn’t know this information would be required at a later stage in my life*” (male, 63 years old).

Some respondents, however, perceived some sexual behaviours as either socially acceptable or unacceptable:

“*I didn’t have a problem to answer [meaning age at first sex]…I think I was at the right age to have sex*” (male, 42 years old).

“*It was difficult to answer this question [about anal sex]. It [anal sex] is for homosexuals…and practiced in prisons*” (male, 34 years old).

Respondents did not generally find it difficult to recall details of specific sexual relationships, especially when discussing current sexual relationships which were going well. However a small number of participants found the partner-specific section difficult because it was depressing to talk about ex-partners; this suggests that participants may differentially underreport relationships that are concluded or undergoing strain:

“*I felt unhappy…I didn’t really love one of them [meaning sexual partner]*” (female, 51 years old).

Furthermore, a 75 year old female respondent repeatedly stated that she felt uncomfortable answering many questions about her sexual behaviour from the distant past with a much younger interviewer.

Respondents were also aware that reporting multiple recent partners might lead to more questions or more complex cognitive processes, with some commenting on their relief that they had few partners to report.

#### Differences from previous surveillance interviews

Half of those respondents who had previously completed AHRI sexual health questionnaires in annual surveillance using PAPI methods found it easier than before. The current version was seen as easier due to: (i) similar question wording to previous questionnaires; (ii) non-inclusion of more sensitive questions (e.g. self-reported HIV status); and (iii) the use of tablet computers. Amongst those in self-interview arms, the explicit option to not answer each question was appreciated. The other half of repeat respondents found the questionnaire harder than before, due to: (i) increased questionnaire length; (ii) perceived repetition of questions; and (iii) difficulty of recall, especially for older respondents.

The majority of participants had positive comments regarding the use of a computer in the EDM interview, such as “felt comfortable”, “felt no problem”, “felt good”, “happy about the computer”, “felt at ease after the practice”, “easy to use computer”, “comfortable with technology” and “happy about self-interview”.

#### Benefits and drawbacks of using electronic delivery methods

Tablets were seen as making interviews quicker and simpler than paper-based forms, as well as increasing confidentiality, trust and security – particularly for the self-interview arms.

*“The use of computers made it easier…in the past [AHRI] used paper-based questionnaires, which compromised confidentiality. Interviewers could disclose our information to other people…but the use of computers protects our information”* (Male, 29 years old, CAPI).

*“No one can see our information on the tablet but paper questionnaires might get lost and found by other people who then read our confidential information”* (Female, 20 years old, CAPI).

Participants in the self-interview arms broadly expressed excitement and comfort about answering questions themselves on the computer. However, some respondents reported that the self-interview methods placed more demand on the participant, since reading questions requires attention and focus; furthermore, one respondent, a 37 year old man, reported that the ACASI method felt slow.

In addition, some participants also expressed concerns about the use of tablets due to illiteracy, having lower education levels, or having eyesight problems.

The group discussion with study interviewers reinforced several themes from the cognitive interviews. These themes included respondent perceptions that self-interview methods were exciting and more confidential, although these factors led to slower interviews. Additionally, interviewers reported that self-interviews increased respondent trust in interviewers and the research process, since respondents had previously thought interviewers were making up some questionnaire questions (especially on sensitive topics), but now they could see that interviewers had not been misleading them. Interviewers also reported their preference for CAPI over other methods, since it was the fastest of all four methods, much lighter than carrying paper, and helped ensure data quality through skip patterns and error warnings.

## Discussion

In this study, we found that the use of electronic delivery methods, including self-interview approaches, was broadly feasible and acceptable in rural South Africa, across a wide range of interviewees. Additionally, while self-interview methods did not consistently impact the rate at which sexual behaviours were reported, they did increase the level of reporting for sexual behaviours most likely to suffer from social desirability bias. Whilst this increase was small in absolute terms (approximately 2 percentage points) it reflected a 45% relative increase in reporting rates. Self-interviews also increased item non-response rates by a similar absolute and relative amount. The study finds that there were both advantages and disadvantages to using self-interviews in this setting.

The great majority of respondents who were offered the opportunity to self-interview did so. Amongst the subsample invited to discuss their experiences, the great majority expressed positive feelings about the interview process and the use of electronic and self-interview methods. Furthermore, the study fieldworkers reported that the CAPI software reduced the risk of data entry inconsistencies and errors. Several of the respondents aged over 30 declined a self-interview due to limited literacy or vision, although this was much reduced in the audio self-interview (ACASI) arm. However, the ACASI interviews were significantly slower to complete, potentially due to the novelty of listening to questions on headphones.

We did not find significant differences in willingness to participate in the study by arm, potentially due to very high response rates in all arms. Response rates were substantially higher for this trial than for the annual surveillance conducted in the same population. These higher response rates may have been due to the perceived novelty of the trial, particularly since the AHRI-standard community engagement “roadshows” held in each trial area 1 week prior to EDM interviews taking place appeared to generate substantial interest in the study: several respondents mentioned these roadshows to interviewers.

Rates of item non-response, i.e. opting out of questions, were frequently higher in self-interview arms, especially for detailed questions about sexual behaviour with MRP and for receipt of support and forced sex questions; item non-response was lower in self-interview arms for age-related and condom use questions, and for anal sex. Past literature suggests we might expect higher rates of non-response in self-interviews for questions requiring complex thought – either to understand or recall – and lower rates for more sensitive topics [16, 19]. Our findings do not firmly support these patterns.

Reporting of sensitive or socially undesirable behaviours differed less across study arms than has been seen in other similar studies [11, 15]; our work was powered to see differences of 10 percentage points for sensitive questions, rather than the 2 percentage point difference we saw on average. This smaller difference may reflect a truly relatively low-risk sexual behaviour profile in this community, or the impact of self-interview privacy may be limited in this rural, African setting: in a recent meta-analysis of self- vs. face-to-face-interviews, Phillips and colleagues saw greater differences in urban, higher-educated and Asian populations [11].

Alternatively, it may be that study participants in this population have learned how to rapidly negotiate structured questionnaires so as to minimize their response burden [5], while still complying with the request to participate due to extrinsic motivation (either controlled – to avoid shame/guilt – or autonomous – because they see responding as important to society) [36]. In such a scenario, while a novel delivery method providing greater privacy might induce some respondents to provide a fuller picture of their sexual history, most respondents will continue to follow the response script that they have developed previously. Such an interpretation is supported by cognitive interview responses implying awareness that reporting more than one sexual partner would lead to additional follow-up questions. The lack of significant within-individual change from previous surveillance questionnaires to this EDM questionnaire for time-invariant questions also lends some support to the idea that the EDM trial may not have strongly affected willingness to report sensitive information. This study cannot directly confirm such a “scripting” explanation, but does suggest that future in-depth interviews might fruitfully investigate this possibility.

Nevertheless, the response pattern in this study does suggest two, countervailing, trends which highlight the trade-offs of using self-interview methods in this setting. First, some sensitive questions (e.g. >1 partner in the past year, recent non-conjugal partners, history of exchange sex, history of anal sex) were answered affirmatively more often in self-interview arms. Within the self-interview arms a few outlier responses were provided (e.g. one respondent reported eight partners in the past year and current involvement with six). Second, there were higher rates of item non-response in self-interview arms, especially for sensitive and partner-specific questions. This latter is likely to reflect the on-screen option to skip any question by choosing “prefer not to answer”, which was not presented explicitly to the respondent in interviewer-led interviews. The combination of these trends suggests that self-interviews are likely to increase reporting of sensitive events, at the cost of higher missingness that is likely to be differential by respondent characteristics.

The decision as to whether to use self-interviews in a particular context will depend on whether the expected advantages outweigh the potential disadvantages of the self-interview method in a given setting. Specifically, if (computer) literacy in the research population is high enough, the research topic sufficiently sensitive, and the expected or pilot-tested increase in response rates elicited by self-interview methods substantial, then it may be worth the additional time taken to complete questionnaires using self-interviews. This approach may address possible biases introduced by higher non-participation due to limited literacy in some subgroups, and higher item non-response by those with behaviours they are unwilling to acknowledge or report.

### Strengths and limitations

This study benefited greatly from a very well-defined population base arising from repeated censuses of the study area, from which a truly random sample could be drawn. The conduct of interviewer-led interviews by local residents with substantial experience of answering similar questionnaires ensured that the comparison between interviewer- and self-led interviews was a fair one of the strongest available version of each method. One limitation of the study was that sampled residents were informed as to their study arm assignment prior to inviting them to participate, potentially biasing response rates; however, very few people declined to participate and thus this issue is unlikely to have had substantive impact. As ever, reporting of sexual behaviours is hard to validate, and so we cannot test which responses were in fact closest to the gold standard of actual activity.

## Conclusion

Electronic data collection methods, including self-interview methods, appear to be feasible and acceptable in a poor, rural South African setting. The use of computer-based self-interviews is likely to become even more feasible as smartphone penetration rises and an increasing proportion of the population are members of younger “digital native” cohorts. However, the use of such methods in place of paper-based approaches did not substantially change the data provided by respondents. Furthermore, self-interview methods provided respondents with greater ability to skip questions which they were uncomfortable answering. Interviewers considering using electronic or self-interview methods should carefully consider the relative benefits and costs of such approaches in their specific context.

## Additional file

Additional file 1: Additional analyses and materials. (PDF 692 kb)

## Abbreviations

ACASI: Audio computer-assisted self-interview
AHRI: Africa Health Research Institute
AT: As treated
CAPI: Computer-assisted personal interview
CASI: Computer-assisted self-interview
DSA: Demographic surveillance area
EDM: Electronic delivery methods
HIV: Human immunodeficiency virus
IQR: Interquartile range
ITT: Intention-to-treat
MRP: Most recent partner
PAPI: Paper and pen interview
SPM: Single-paper meta-analysis
