# Supplementary material for: The impact of self-interviews on response patterns for sensitive topics: a randomized trial of electronic delivery methods for a sexual behaviour questionnaire in rural South Africa
Source: BMC Med Res Methodol. 2017 Aug 17;17:125. doi: 10.1186/s12874-017-0403-8 (PMC5561578; doi:10.1186/s12874-017-0403-8)
Supplement: Additional file 1: — Additional analyses and materials. (PDF 692 kb) [file 12874_2017_403_MOESM1_ESM.pdf]

Supplementary Material

Title: The impact of self-interviews on response patterns for sensitive topics: a randomized trial of electronic delivery methods for a sexual behaviour questionnaire in rural South Africa

Supplementary Content 1: Cognitive interview questions: general probes only

All interviews were conducted entirely in isiZulu. Cognitive interview prompts were asked at the end of each section of the interview. The prompts shown here are only those that are not specific to any one question and refer to the sexual behaviour sections of the questionnaire.

<u>Section 7: General sexual history, part 1</u>

7.1 How did you find it answer those questions about your sexual history?

7.2 How did you feel about answering those questions?

<u>Section 8: Partner-specific sexual history</u>

8.1 How did you find it answer those questions about your recent partners?

8.2 How did you feel about answering those questions?

8.3 How easy or difficult was it for you to identify your most recent partners?

8.4 How did you feel about giving us nicknames for your most recent partners?

<u>Section 9: General sexual history, part 2</u>

9.1 How did you find it answer those questions about your sexual history?

9.2 Did you find that section embarrassing to answer?

<u>Section 10: Overall</u>

10.1 How easy or difficult was it to answer the whole questionniare?

10.2 Compared to previous times that you have answered Africa Centre questionnaires about sexual behaviour, was it easier or harder than those times?

10.3 Compared to previous times, how did it feel this time?

10.4 Are there any other questions about sexual behaviour that you think the Africa Centre should be asking?

CAPI/CASI/ACASI only:

10.5 How did you feel about the use of a computer in this interview?

CASI/ACASI only:

10.6 How did you feel about answering questions yourself on the computer?

10.7 Was the practice self-interview helpful?

Supplementary Content 2: Comparative analysis of EDM and surveillance responses

Methods

In addition to conducting cross-sectional analysis, we also compared individuals' responses in this trial to their most-recent responses in a surveillance questionnaire. This supplementary analysis aimed to evaluate to what extent results seen in the EDM trial reflected the trial environment itself: i.e. if those in the interviewer-led arms responded differently in the EDM versus in surveillance. We achieved this by matching each individual to their most recent surveillance questionnaire response, based on the last time they consented to respond to sexual behaviour questions. Since surveillance questionnaires for our trial sample are elicited between January and May each year, the minimum possible time since last surveillance response was approximately 6 months. We then compared response rates across interviewer- and respondent-led arms for four key questions: (i) ever had sex; (ii) age of sexual debut; and number of sexual partners in (iii) lifetime; and (iv) the past 12 months. We expect questions (i) and (ii) to be invariant for any respondent who reported ever having had sex in the earlier surveillance. We expect questions (iii) and (iv) to rise for some respondents between interviews, with an increasing probability as the duration between questionnaires increases. We again used χ^2 tests for binary outcomes and Kruskal-Wallis tests for ordinal outcomes, and Wilcoxon rank-sum tests for continuous outcomes.

Results

Of the 340 participating individuals, 24 (7.1%) had never completed an individual surveillance questionnaire, 35 more (10.3%) had never answered sexual health questions

before and a further 27 (8.0%) had never answered any sexual behaviour questions before. Of the remaining 254 respondents who had ever answered sexual behaviour questions, the median last sexual behaviour questionnaire was in 2013 (IQR: 2010-2014); 155 had participated in the general health questionnaire earlier in 2015, but only 68 had completed the sexual health, and 54 the sexual behaviour, modules.

We compared responses in this EDM questionnaire to those in the most-recent surveillance questionnaire in which respondents answered sexual behaviour questions. The sample for these analyses was the 254 individuals who completed our EDM questionnaire and had ever previously provided responses on their sexual behaviour; none of the results changed when we restricted our analysis to the 54 individuals who provided sexual behaviour surveillance responses in 2015. The median time between most-recent sexual behaviour questionnaire and EDM interview was 2.6 years (IQR: 1.5-5.4) as shown in Supplementary Figure 1. Amongst the 34 individuals who had reported never having had sex at their last surveillance interview (10 of 13 women and 17 of 21 men were aged under 30), 20 (7 women, 13 men) reported having had sex in the EDM (median time between questionnaires: 1.5 years, IQR 0.5-2.6). There was no statistically significant difference between those in the personal-interview and self- interview arms ($\chi_1^2 = 0.01$, p=0.93). One 28 year-old man and one 31 year-old woman who previously reported having had sex, reported never having had sex in the EDM.

203 respondents who had previously completed a sexual behaviour questionnaire reported their age at first sex in the EDM. Of these, 120 (59.1%) had reported their age at first sex in their most recent preceding questionnaire (of the others, 23% reported never having had

sex and 71% could not recall their age at first sex). Of the 120, the median change in age of debut by the EDM was zero – with 78% reporting an age within two years of their previous response. These values did not significantly vary by trial arm (Wilcoxon $Z = 0.04$, p=0.97) or gender ($Z = 1.53$, p=0.12). The EDM-reported age of sexual debut for these 120 did not significantly differ from the 83 individuals who did not provide an age previously or the 66 individuals provided their age at first sex in the EDM but who had not previously completed a sexual behaviour questionnaire (Kruskal-Wallis $\chi_2^2 = 1.99$, p=0.37).

Among the 169 EDM participants who most recently completed a surveillance questionnaire after 2011, when this question began to be asked, 101 of the 135 who reported having had sex by that questionnaire (74.8%) reported their lifetime number of sexual partners in their most recent surveillance questionnaire. By the EDM interview, 154 of the 169 reported ever having had sex and 128 (83.1%) reported a lifetime number of partners. Non-reporting rates were higher for men for both surveillance and EDM interviews, and fell by approximately one-third for both genders from last surveillance to EDM (from 19% to 12% for women; from 34% to 23% for men). Among the 84 respondents answering on both occasions, the mean number of partners reported rose from 1.9 to 3.7 and the maximum from 6 to 25 (one 34 year-old man reported 50 partners having previously declined to respond). However, although the rise in partner numbers reported was lower for PAPI compared to all other arms (IQR increase 0-1 vs. 0-2; mean increase 0.45 vs. 2.2 partners), there was no significant difference either between personal-interview and self-interview arms (Wilcoxon: $Z = 1.03$, p=0.30) or between PAPI and all other arms combined ($Z = 1.07$, p=0.28). Similarly, while men reported a larger rise than women (mean increase 3.9 vs. 0.7) and those aged under 30 a larger rise than those aged

30-49 or $\geqslant$50 (mean increase 2.2 vs. 1.1 and 1.8), these differences were not statistically different (Wilcoxon $Z = 1.71$, p=0.09 and Kruskal-Wallis$\chi^2_2 = 2.90$, 0.23 respectively).

207 EDM participants had previously reported their number of sexual partners in the past 12 months; 164 of these provided a number for the corresponding EDM question. Previously none of these individuals has reported more than two partners in the past 12 months; in the EDM four (three men in their 20s and a woman aged 30) individuals reported three partners and one person (a 35 year-old man) reported eight. There was no significant difference in change in number of sexual partners between personal-interview and self-interview arms (Wilcoxon $Z = 1.21$, p=0.23).

Supplementary Table 1: Study outcomes for sampled individuals

		Study arm				
		PAPI	CAPI	CASI	ACASI	Total
Initial allocation		*126*	*125*	*127*	*126*	*504*
	Temporarily away	6	8	2	8	24
	Out-migrated	11	14	14	16	55
	Deceased	1	0	2	2	5
Present in DSA		*108*	*103*	*109*	*100*	*420*
	Not found	15	14	13	13	55
Found		*93*	*89*	*96*	*87*	*365*
	Unable to consent	2	3	8	2	15
Asked to consent		*91*	*86*	*88*	*85*	*350*
	Declined to interview	1	2	2	5	10
Interviewed ITT		*90*	*84*	*86*	*80*	*340*
	Switched to CAPI			24	5	29
Interviewed AT		*90*	*113*	*62*	*75*	*340*

PAPI: Paper and pen interview; CAPI: computer-assisted self-interview; CASI: computer-assisted personal interview; ACASI: audio computer-assisted personal interview. ITT: Intention-to-treat; AT: As treated.

Supplementary Figure 1: Distribution of most recent response to sexual behaviour surveillance questionnaire

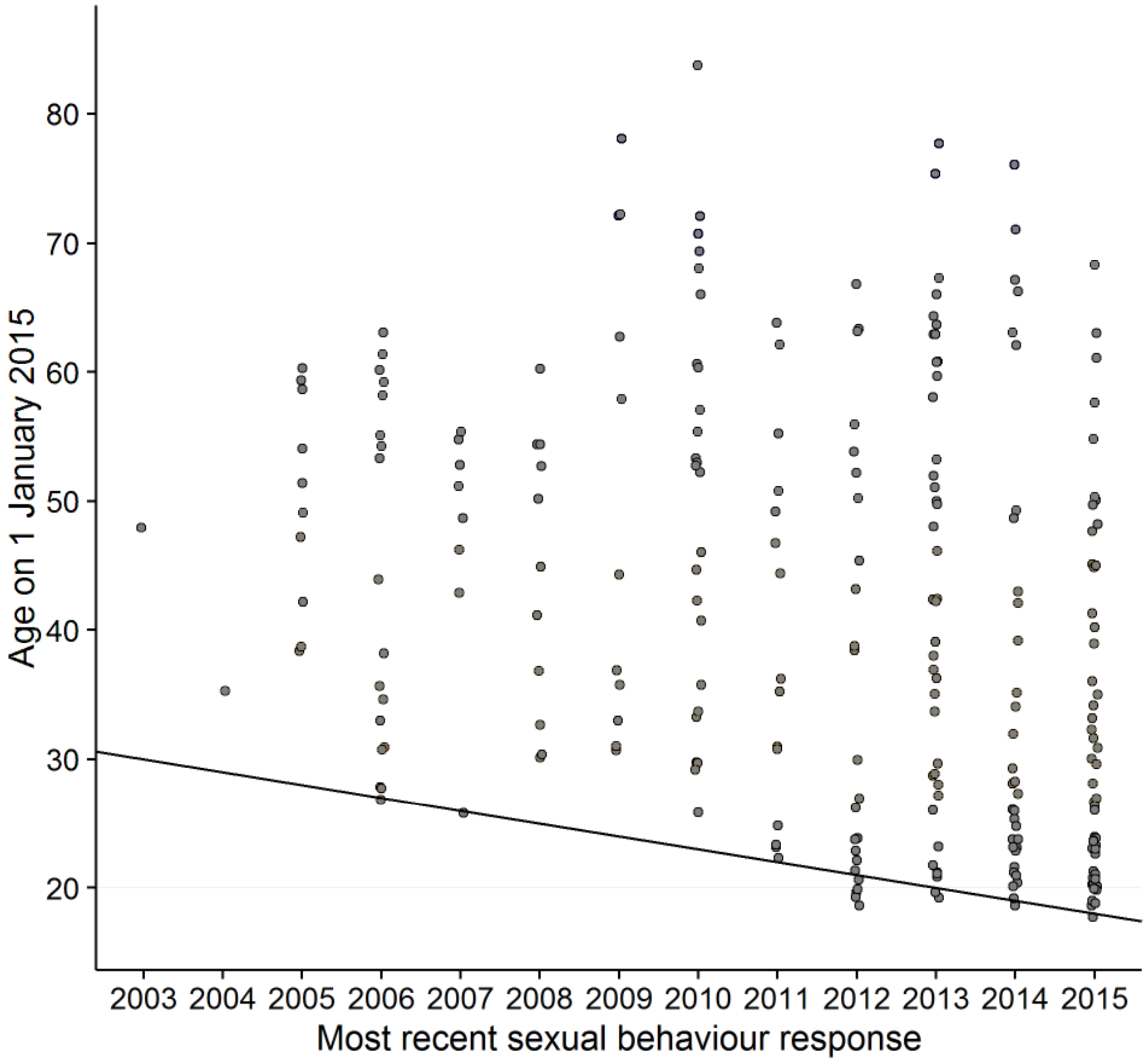

N=254. Distribution of most-recent surveillance questionnaire response vs. age at start of 2015. Individuals on the diagonal line are age 18 in each year.

Supplementary Figure 2: Single-paper meta-analysis of item non-response for all sexual behaviour questions

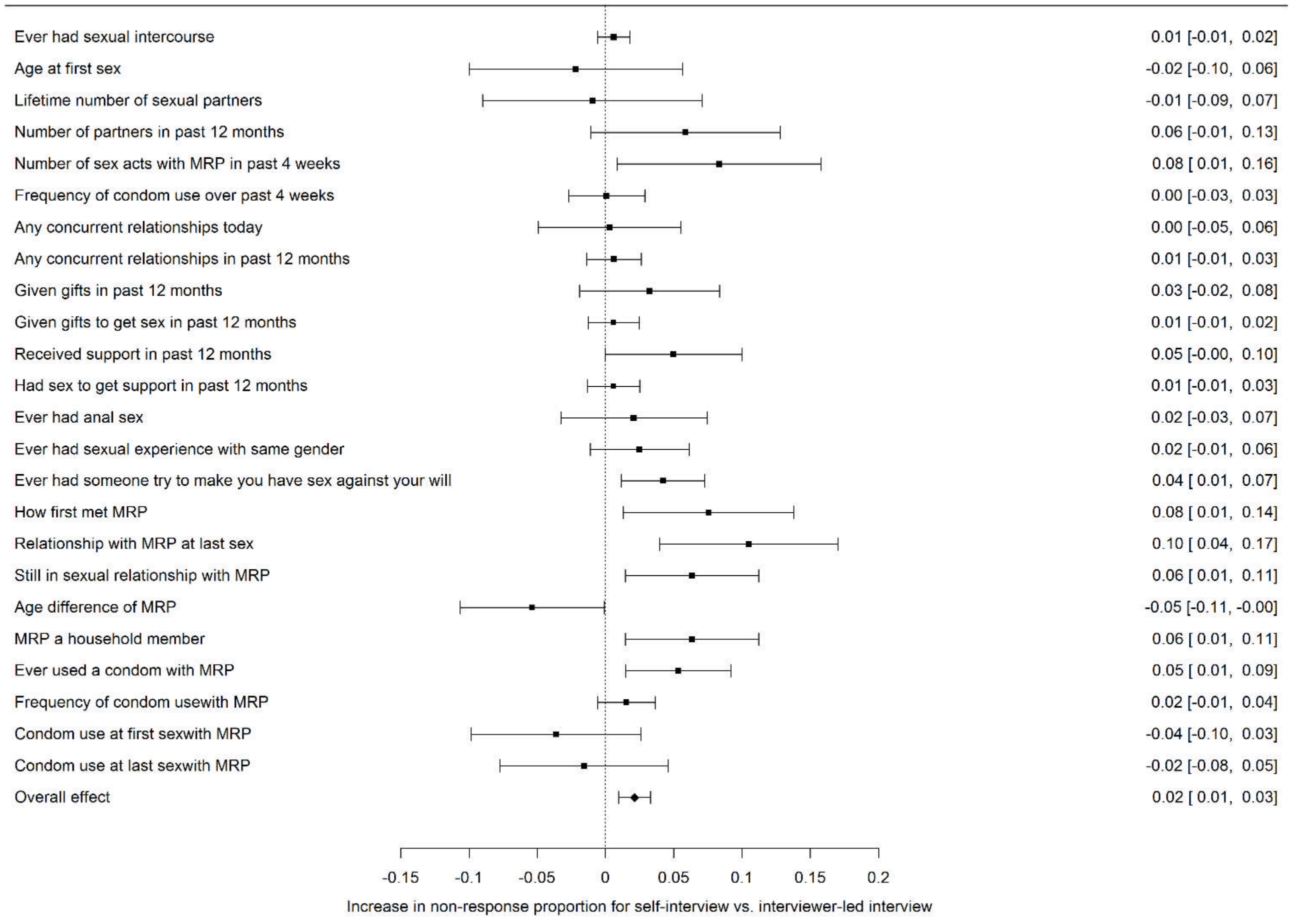

Size of point estimates is in proportion to the log of the number of respondents for each question. Values at right are means and 95% confidence intervals. Overall the mean item non-response rate was 4.4% for interviewer-led arms vs. 6.5% for self-interview arms (difference 2.1%, 95% confidence interval: 0.1 – 3.3%). Heterogeneity was very high: I^2=88.4, 95% confidence interval: 85.4-90.7%.

Supplementary Figure 3: Single-paper meta-analysis of all binary response sexual behaviour questions

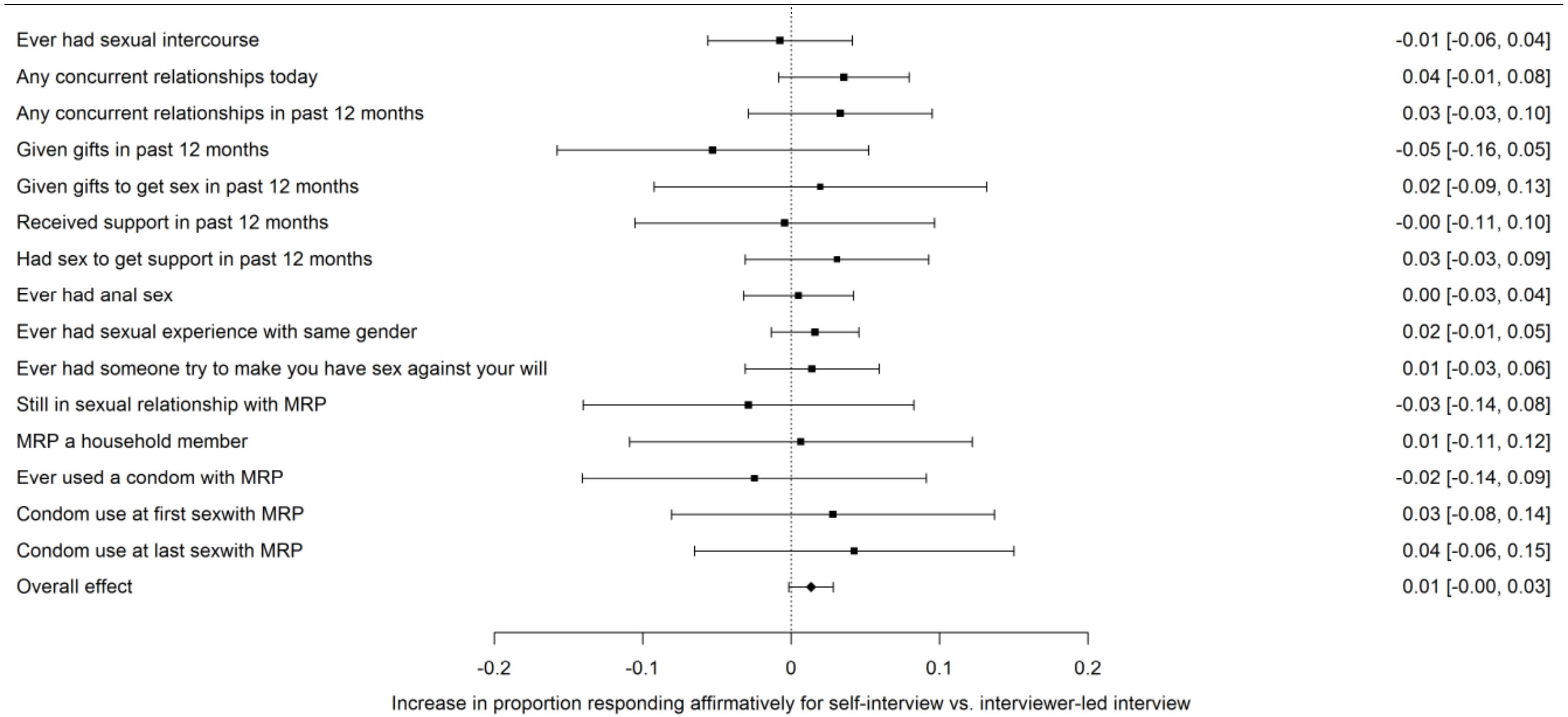

Size of point estimates is in proportion to the log of the number of respondents for each question. Values at right are means and 95% confidence intervals. Overall the mean affirmative response rate was 27.8% for interviewer-led arms vs. 29.1% for self-interview arms (difference 1.3%, 95% confidence interval: -0.1 – 2.8%). Heterogeneity was extremely high: I^2=99.50, 95% confidence interval: 99.45-99.55.
